# Development of a microRNA Panel for Classification of Abnormal Mammograms for Breast Cancer

**DOI:** 10.3390/cancers13092130

**Published:** 2021-04-28

**Authors:** Ruiyang Zou, Sau Yeen Loke, Veronique Kiak-Mien Tan, Swee Tian Quek, Pooja Jagmohan, Yew Chung Tang, Preetha Madhukumar, Benita Kiat-Tee Tan, Wei Sean Yong, Yirong Sim, Sue Zann Lim, Eunice Png, Shu Yun Sherylyn Lee, Mun Yew Patrick Chan, Teng Swan Juliana Ho, Boon Kheng James Khoo, Su Lin Jill Wong, Choon Hua Thng, Bee Kiang Chong, Yik Ying Teo, Heng-Phon Too, Mikael Hartman, Ngiap Chuan Tan, Ern Yu Tan, Soo Chin Lee, Lihan Zhou, Ann Siew Gek Lee

**Affiliations:** 1 Department of Research and Development, MiRXES Lab, Singapore 138623, Singapore; ruiyang_zou@mirxes.com (R.Z.); yewchungtang@mirxes.com (Y.C.T.); 2 Cellular and Molecular Research, Humphrey Oei Institute of Cancer Research, National Cancer Centre Singapore, Singapore 169610, Singapore; loke.sau.yeen@gmail.com; 3 SingHealth Duke-NUS Oncology Academic Clinical Programme, Duke-NUS Medical School, Singapore 169857, Singapore; veronique.tan.k.m@singhealth.com.sg (V.K.-M.T.); madhukumar.preetha@singhealth.com.sg (P.M.); benita.tan.k.t@singhealth.com.sg (B.K.-T.T.); yong.wei.sean@singhealth.com.sg (W.S.Y.); sim.yirong@singhealth.com.sg (Y.S.); lim.sue.zann@singhealth.com.sg (S.Z.L.); juliana.ho.t.s@singhealth.com.sg (T.S.J.H.); james.khoo.b.k@singhealth.com.sg (B.K.J.K.); thng.choon.hua@singhealth.com.sg (C.H.T.); 4 National Cancer Centre Singapore, Division of Surgery and Surgical Oncology, Singapore 169610, Singapore; 5 Department of Breast Surgery, Singapore General Hospital, Singapore 169608, Singapore; 6 SingHealth Duke-NUS Breast Centre, Singapore 169610, Singapore; 7 Departments of Diagnostic Imaging, National University Hospital and Yong Loo Lin School of Medicine, National University of Singapore, Singapore 119228, Singapore; dnrqst@nus.edu.sg (S.T.Q.); jagmohan_pooja@nuhs.edu.sg (P.J.); 8 Department of General Surgery, Sengkang General Hospital, Singapore 544886, Singapore; 9 SingHealth Polyclinics, Singapore 150167, Singapore; eunice.png.s.y@singhealth.com.sg (E.P.); tan.ngiap.chuan@singhealth.com.sg (N.C.T.); 10 Department of General Surgery, Tan Tock Seng Hospital, Singapore 308433, Singapore; sherylyn_lee@ttsh.com.sg (S.Y.S.L.); patrick_chan@ttsh.com.sg (M.Y.P.C.); ern_yu_tan@ttsh.com.sg (E.Y.T.); 11 National Cancer Centre Singapore, Division of Oncologic Imaging, Singapore 169610, Singapore; jill.wong.s.l@singhealth.com.sg; 12 Department of Diagnostic Radiology, Tan Tock Seng Hospital, Singapore 308433, Singapore; chong_bee_kiang@ttsh.com.sg; 13 Saw Swee Hock School of Public Health, National University of Singapore and National University Health System, Singapore 117549, Singapore; yyteo@nus.edu.sg (Y.Y.T.); mikael_hartman@nuhs.edu.sg (M.H.); 14 Department of Biochemistry, Yong Loo Lin School of Medicine, National University of Singapore, Singapore 119077, Singapore; bchtoohp@nus.edu.sg; 15 Department of Surgery, Yong Loo Lin School of Medicine, National University of Singapore and National University Health System, Singapore 119228, Singapore; 16 SingHealth Duke-NUS Family Medicine Academic Clinical Programme, Duke-NUS Medical School, Singapore 169857, Singapore; 17 Department of Hematology-Oncology, Yong Loo Lin School of Medicine, National University of Singapore and National University Health System, Singapore 119228, Singapore; soo_chin_lee@nuhs.edu.sg; 18 Department of Physiology, Yong Loo Lin School of Medicine, National University of Singapore, Singapore 117593, Singapore

**Keywords:** breast cancer, abnormal mammograms, circulating microRNAs, biomarkers, qRT-PCR

## Abstract

**Simple Summary:**

Breast cancer screening by mammography suffers from high rates of false positivity, resulting in unnecessary investigative imaging and biopsies. There is an unmet need for biomarkers that can distinguish between malignant and benign breast lesions. We performed miRNA profiling on 638 patients with abnormal mammograms and 100 healthy controls. A six-miRNA panel was identified and validated in an independent cohort that had an AUC of 0.881 when differentiating between cases versus those with benign lesions or healthy individuals with normal mammograms. In addition, biomarker panel scores increased with tumor size, stage and number of lymph nodes involved. This study demonstrates that circulating miRNAs can potentially be used in conjunction with mammography to differentiate between patients with malignant and benign breast lesions.

**Abstract:**

Mammography is extensively used for breast cancer screening but has high false-positive rates. Here, prospectively collected blood samples were used to identify circulating microRNA (miRNA) biomarkers to discriminate between malignant and benign breast lesions among women with abnormal mammograms. The Discovery cohort comprised 72 patients with breast cancer and 197 patients with benign breast lesions, while the Validation cohort had 73 and 196 cancer and benign cases, respectively. Absolute expression levels of 324 miRNAs were determined using RT-qPCR. miRNA biomarker panels were identified by: (1) determining differential expression between malignant and benign breast lesions, (2) focusing on top differentially expressed miRNAs, and (3) building panels from an unbiased search among all expressed miRNAs. Two-fold cross-validation incorporating a feature selection algorithm and logistic regression was performed. A six-miRNA biomarker panel identified by the third strategy, had an area under the curve (AUC) of 0.785 and 0.774 in the Discovery and Validation cohorts, respectively, and an AUC of 0.881 when differentiating between cases versus those with benign lesions or healthy individuals with normal mammograms. Biomarker panel scores increased with tumor size, stage and number of lymph nodes involved. Our work demonstrates that circulating miRNA signatures can potentially be used with mammography to differentiate between patients with malignant and benign breast lesions.

## 1. Introduction

Breast cancer is the most commonly diagnosed cancer and the leading cause of cancer mortality among women worldwide [1]. The gold standard for breast cancer screening is mammography [2]. However, this modality yields significant false-positive results that require additional diagnostic imaging procedures and tissue biopsies [2]. In a large study of 702,154 cases in the United States, 171,829 had abnormal screening mammogram results with only <2% (2599/171,829) found to be true positives [3]. As a result, a large majority of these women with abnormal screening mammograms were subjected to invasive and expensive diagnostic procedures that could have been avoided. Thus, there is a need to develop a more accurate breast cancer detection modality for this prevalent malignancy among women.

In recent years, multiple advanced imaging methods and non-invasive biomarkers have been developed for improving breast cancer detection [4,5]. In particular, liquid biopsies have revolutionized cancer diagnostics, therapeutics and monitoring by offering a minimally invasive clinical modality that is reliable for real-time personalized molecular profiling [6]. It harnesses the analysis of circulating biomolecules in the blood, including circulating tumor cells (CTCs), circulating cell-free DNA (cfDNA), circulating microRNAs (miRNAs) and tumor-derived extracellular vesicles (EVs), to evaluate the presence or progression of cancer [6].

Circulating miRNAs were first reported in 2008 as potential biomarkers for solid cancers [7]. MiRNAs are single-stranded non-coding RNAs with 19 to 25 nucleotides that play important roles in the translational repression or degradation of mRNA targets [8]. Circulating miRNAs have been deemed suitable as cancer biomarkers because of their stability in various body fluids and their differential expression profiles during tumor progression [8,9]. Their potential clinical application for diagnosis, prognosis, monitoring and therapy has been demonstrated in a wide range of cancers such as gastric, colorectal, liver, pancreatic, lung, cervical, prostate and breast cancers [10]. In breast cancer, various potential serum and plasma miRNA biomarkers have been reported [11]. Several studies have identified circulating miRNA signatures for breast cancer with AUCs of 0.700 and above, in various cohorts of Caucasian and Asian populations [12,13,14,15,16,17,18,19,20,21,22]. More recently, the first circulating eight-miRNA signature for breast cancer derived from a next-generation sequencing transcriptome analysis was reported [23]. Despite the various promising findings for breast cancer circulating miRNAs, not all biomarker candidates had been validated in independent validation cohorts, with none yet proceeding to clinical studies [11,14,18,19,22,24,25].

In the present study, miRNA profiling using qRT-PCR was used to discover a panel of serum miRNAs for the stratification of malignant and benign breast lesions. Our study design included independent Discovery and Validation cohorts (*n* = 269 for each cohort) with a biomarker panel built using a two-fold cross-validation procedure, incorporating a feature selection algorithm and a logistic regression predictive model.

## 2. Results

### 2.1. Identification of miRNAs Expressed in Malignant and Benign Breast Lesions

Candidate serum miRNA biomarkers for differentiating between patients with benign breast lesions and those with breast cancer were identified from analysis of the Discovery cohort (Table 1). Absolute expression levels of 324 miRNAs were profiled in 72 breast cancer patients and compared to those in 197 patients with benign breast lesions. Among the 324 miRNAs analyzed, 179 miRNAs were found to be expressed at 500 or more copies per ml of serum in all subjects.

### 2.2. Identification and Evaluation of miRNA Biomarker Signatures Using Three Strategies

#### 2.2.1. Differentially Expressed Individual miRNA Biomarkers

**Figure 1:**
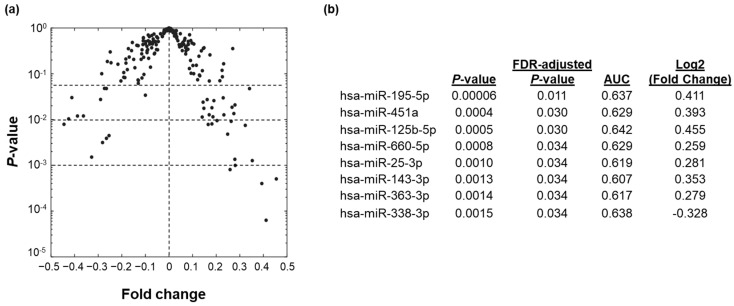
Biomarker discovery. (**a**) Differential miRNA expression in patients with breast cancer compared to those with benign breast lesions as determined by fold change of miRNA expression and statistical significance (*p*-value). (**b**) Differentially regulated miRNAs in patients with breast cancer compared to those with benign breast lesions (statistical significance determined by FDR-adjusted *p*-value < 0.05). Ability of miRNA biomarker in distinguishing between breast cancer and benign breast lesions was measured by area under the ROC curve (AUC).

Three strategies were used to identify and evaluate miRNA biomarker signatures that could distinguish between malignant and benign breast lesions (graphical abstract). Firstly, we identified miRNAs which were differentially expressed between malignant and benign breast lesions based on fold-change of normalized miRNA expression and statistical testing. Among the 179 miRNAs expressed, 8 miRNAs were found to be either significantly higher (7 miRNAs with FDR-adjusted *p* < 0.05) or lower (1 miRNA with FDR-adjusted *p* < 0.05) in breast cancer cases as compared to those with benign breast lesions (Figure 1). All 8 of these differentially regulated miRNAs could differentiate breast cancer from benign breast lesions with AUC of 0.607 to 0.642 when used individually as biomarkers (Figure 1b). No miRNAs were found to be differentially expressed between the various molecular subtypes of breast cancer (estrogen receptor-positive, progesterone receptor-positive, and HER2 overexpressed) nor did any miRNA perform better as a biomarker for a certain molecular subtype.

#### 2.2.2. Multi-miRNA Biomarker Panels Built through a Focused Search

**Figure 2:**
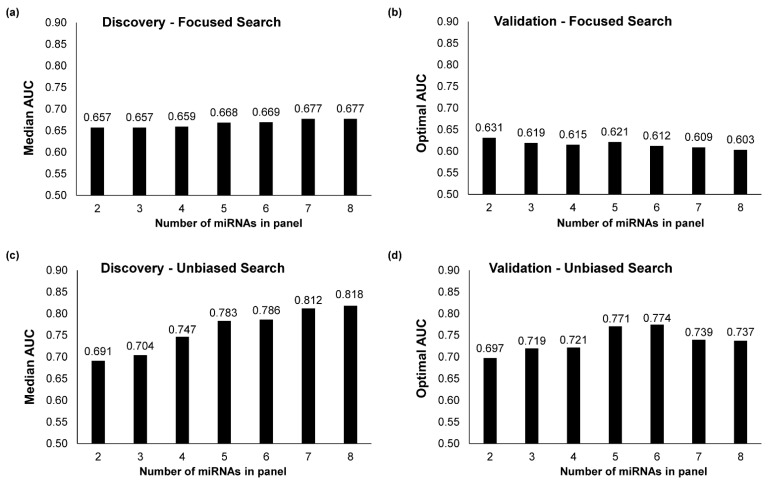
Biomarker panel building. Median AUC values for multi-miRNA biomarker panels identified through (**a**) a focused search and (**c**) an unbiased search in the Discovery Cohort. AUC values for optimal multi-miRNA biomarker panels from the (**b**) focused search and (**d**) unbiased search validated in the Validation cohort. The focused search refers to a search among the 8 top-ranked differentially regulated miRNAs only while the unbiased search expanded the search to all 324 miRNAs analysed in this study.

Secondly, we built multi-miRNA biomarker panels comprising 2 to 8 miRNAs by focusing on the 8 individual differentially expressed miRNA biomarkers described above. The biomarker panels were evaluated based on their AUC in distinguishing between malignant and benign breast lesions. AUCs of multi-miRNA biomarker panels built through this focused search showed no significant improvement compared to AUCs of individual miRNA biomarkers (Figure 2a). The maximum AUC was achieved by the focused seven-miRNA and eight-miRNA biomarker panels, each with an AUC of 0.677. Validation of focused multi-miRNA biomarker panels in the Validation cohort confirmed that focusing only on the top differentially expressed miRNAs did not improve biomarker panel performance (Figure 2b).

#### 2.2.3. Multi-miRNA Biomarker Panels Built through an Unbiased Search

Thirdly, we built multi-miRNA panels comprising two to eight miRNAs through an unbiased search among all miRNAs expressed in malignant and benign breast lesions. Through the same cross-validation procedure as in the focused search, we built optimal unbiased two-miRNA to eight-miRNA biomarker panels. Using these unbiased multi-miRNA panels, the maximum median AUC achieved was 0.818, with AUC improving as the number miRNAs in the biomarker panel was increased from two to eight (Figure 2c). When the optimal unbiased multi-miRNA biomarker panels were validated in the Validation cohort, the highest AUC of 0.774 was achieved by an unbiased six-miRNA biomarker panel (Figure 2d).

### 2.3. Selection of Optimal miRNA Biomarker Signature

**Figure 3:**
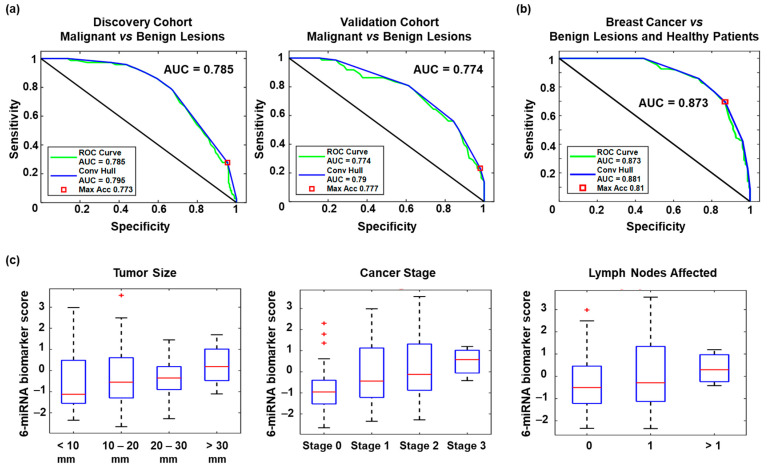
Biomarker panel validation. (**a**) ROC curves for the optimal 6-miRNA biomarker panels used in distinguishing patients with breast cancer from those with benign breast nodule in the Discovery (left panel) and Validation cohort (right panel). (**b**) ROC curves for the optimal 6-miRNA biomarker panels used in distinguishing patients with breast cancer from those with benign breast lesions and normal mammograms. (**c**) Box and whiskers plots of 6-miRNA biomarker scores in differentiating breast cancers by tumor size, stage, and number of lymph nodes affected.

Based on the evaluation of miRNA biomarker signatures identified through the three strategies above, we selected the unbiased six-miRNA biomarker panel as the optimal panel (Table 2) because it had a higher AUC than the 7-miRNA and 8-miRNA panels in validation. The AUC of this selected panel was 0.785 in the Discovery Cohort and 0.774 in the Validation Cohort (Figure 3a). The optimal six-miRNA panel included only 2 miRNAs, miR-195-5p and miR-451a, which were differentially regulated (with FDR-adjusted *p*-value < 0.05) between breast cancer and benign breast lesions. The expression of the six miRNAs were not correlated with breast cancer clinicopathological markers such as estrogen receptor expression, progesterone receptor expression, HER2 overexpression, lymphovascular invasion, and tumour grade.

### 2.4. Performance of the Optimal miRNA Biomarker Signature

Having validated that it was possible to discriminate between breast cancer and benign breast lesions using a six-miRNA biomarker panel with a suitable biomarker score cut-off, we tested patients with normal mammograms using the six-miRNA panel. The six-miRNA biomarker panel had an AUC of 0.881 for identifying patients with breast cancer from those with either benign breast nodules or normal screening mammograms (Figure 3b). From the AUC curve, we identified a high-specificity biomarker score cut-off which gave a specificity of 89.8%, sensitivity of 41.1%, positive predictive value (PPV) of 60%, and negative predictive value (NPV) of 80.4% (Table 3). From the same AUC curve, we also identified an alternative high-sensitivity biomarker score cut-off giving a sensitivity of 79.5%, specificity of 62.2%, PPV of 43.9%, and NPV of 89.1% (Table 3). Among the 145 breast cancer cases in both discovery and validation cohorts, the six-miRNA biomarker scores increased with tumor size, stage, and number of lymph nodes involved (Figure 3c).

## 3. Discussion

The present study describes the discovery of a six-miRNA biomarker panel for breast cancer in the largest cohort of a Southeast Asian population that can discriminate between malignant and benign breast lesions among women with abnormal screening mammograms. The optimal biomarker panel was identified after an evaluation of three different miRNA biomarker signature identification strategies in the Discovery Cohort and was validated in the Validation Cohort. The serum biomarker panel comprising six miRNAs (hsa-miR-451a, hsa-miR-195-5p, hsa-miR-126-5p, hsa-miR-423-3p, hsa-miR-192-5p, and hsa-miR-17-5p) had an AUC of 0.774, demonstrated high negative predictive value (>80%) and produced higher scores with increased tumor size, stage and number of lymph nodes involved. In addition, our results showed that the biomarker panel had a higher diagnostic performance (AUC of 0.881) in differentiating between women with malignant breast lesions versus those with benign breast lesions or healthy women with normal mammograms.

Among the six miRNAs in the panel, three miRNAs, hsa-miR-451a, hsa-miR-195-5p and hsa-miR-126-5p, were significantly upregulated in malignant breast lesions as compared to benign breast lesions. The associations of these miRNAs with breast cancer have also been reported in the literature [26,27,28,29,30,31,32,33]. miR-451a is one of the four miRNAs in a serum miRNA signature that predicts the therapeutic benefit of trastuzumab for HER2-positive metastatic breast cancer patients [26]. miRNA-451a was shown to have primarily originated from the extracellular vesicles of immune cells found in the peripheral blood of five HER2-positive primary breast cancer patients [26]. In addition, this miRNA has been suggested as a potential marker to monitor chemotherapy efficacy for triple-negative breast cancer because the transfection of MDA-MB-231 cells with miR-451a significantly improved the sensitivity of breast cancer cells to doxorubicin [27]. In in vitro experiments, overexpression of miR-451a reduced the expression of target gene macrophage migration inhibitory factor (MIF) and suppressed cell proliferation, colony formation, and invasion of breast cancer cells [28]. This suggests a probable role for the miR-451a/MIF pathway in the biology of breast cancer cells [28].

Similarly, the potential of miR-195-5p for detection or as a therapeutic target for breast cancer has been documented [29,30]. Several studies have suggested the role of miR-195-5p as a tumor suppressor in breast cancer [29,31]. Overexpression of miR-195-5p was found to reduce cell colony formation, inhibit cell proliferation and migration, as well as to cause an accumulation of cells in the G1 phase of the cell cycle [29]. Furthermore, miR-195-5p was one of the four dysregulated miRNAs that was postulated to have an association with HER-2 positive breast cancer trastuzumab resistance [31]. Using pathway mapping tools, the predicted target genes of miR-195-5p have been found to be associated with the PI3K-AKT and MAPK signaling pathways, for drug resistance [31]. In addition, miR-195-5p has been reported to be significantly associated with the ErbB signaling pathway, in a separate study that revealed miR-195-5p as being downregulated in early-stage breast cancer [30].

MiR-126-5p was among the miRNAs that showed reduced expression in triple-negative breast cancer tissues as compared to normal breast tissues [32]. However, a significant linear trend for tumor size value and expression levels of miR-126-5p has been observed where larger tumor size had higher levels of miR-126-5p levels [32]. When comparing blood-derived circulating miR-126-5p expression between patients with basal-like triple-negative breast cancers and healthy controls, miRNA expression was significantly increased before neoadjuvant chemotherapy whereas after the neoadjuvant chemotherapy, expression levels decreased to levels comparable to that of controls [33]. Therefore, circulating miR-126-5p demonstrated a diagnostic potential for triple-negative breast cancer and the analysis of its expression profile changes may be useful in predicting the response to neoadjuvant chemotherapy [33].

There are several strengths in this current study. Firstly, to our knowledge, it is the largest multi-centre cohort study with over 500 individuals prospectively recruited from three tertiary Singapore hospitals and two public primary care clinics and involved independent Discovery and Validation cohorts. Furthermore, stringent measures were taken to minimize confounding factors that might give rise to technical or analytical biasness. For example, in both the Discovery and Validation cohorts, similar sample sizes were used with equal proportions of malignant and benign breast lesions, and factors such as race, tumor stage, size and grade as well as lymph node status were equally distributed in the two cohorts. In addition, despite the prospective recruitment of study subjects and collection of blood samples from different centres, the sample collection, processing, storage, and analysis were consistent at all centres by following a standard protocol. In comparison to other alternatives for profiling miRNA biomarkers such as next-generation sequencing, microarray or NanoString platform, there are various advantages of using a qRT-PCR platform, which has been widely known as the “gold standard” for quantitative analysis of nucleic acids [34]. The qRT-PCR platform that we used in this study is an affordable method that requires small amounts of starting material and can detect miRNA expression at high accuracy and sensitivity with a low detection limit. As compared to other commercially available qRT-PCR platforms, the current one developed by MiRXES is able to yield miRNA copy numbers instead of the usual relative expressions, making it possible to quantitate the absolute expression of miRNAs.

This study had some limitations. Firstly, it is necessary to perform further validation studies in independent cohorts of cases and controls to examine the diagnostic performance of the miRNA biomarker panel, for example in studies with blinded samples. Furthermore, the diagnostic performance of the biomarker panel could be compared with the performance of existing imaging modalities in order to evaluate the potential advantage of incorporating this blood-based biomarker panel into current diagnostic workflows for breast cancer. Secondly, to establish the clinical utility of this miRNA biomarker panel, the performance of the panel could be assessed in blood samples obtained from individuals from other ethnic groups as well as samples from patients diagnosed with other cancers. Thirdly, the sample size for healthy individuals was relatively small as compared to the total sample size of individuals with malignant or benign breast lesions. To overcome this limitation in future studies, an independent cohort of healthy individuals can be included in the training set to generate a new logistic regression model with a better ability to discriminate between malignant cases and non-malignant controls. However, it is important to note that such a diagnostic model would be more suitable for developing a biomarker panel for screening purposes, rather than solely for the stratification of breast lesions with abnormal screening mammograms.

## 4. Materials and Methods

### 4.1. Patient Cohort

This study included 597 patients who had abnormal mammograms detected at 3 sites in Singapore, namely the National University Hospital (NUH), Tan Tock Seng Hospital (TTSH), and National Cancer Centre Singapore (NCCS), between 2016 and 2018. Singapore has a multi-ethnic population comprising of Chinese, Malay, Indian and other ethnic groups, with Chinese making up the majority at 74%. Clinicopathological characteristics of the patients are shown in Table 1. Peripheral blood samples were collected prior to biopsy or surgery. Of these 597 patients, 166 were confirmed to have breast cancer (malignant breast lesions) upon histopathological examination. A total of 59 samples (38 from patients with benign breast lesions and 21 from patients with malignant breast lesions) were excluded from the analysis due to sample hemolysis in the sample, resulting in a total of 393 samples from patients with benign breast lesions and 145 samples from patients with malignant lesions being utilized. Hemolysis causes contamination of serum miRNA with red blood cell miRNA in the sample. In total, 538 samples passed quality control. These samples were equally divided into a Discovery Cohort of 197 patients with benign breast lesions and 72 patients with breast cancer, and a Validation Cohort of 196 cases with benign breast lesions and 73 breast cancer cases. For normal controls, we recruited and collected blood samples from 100 patients who had normal screening mammograms from routine screening at SingHealth Polyclinics. The study was approved by Institutional Review Boards at all study sites and written informed consent was obtained from all study participants.

### 4.2. Blood Collection and Serum Processing

Peripheral blood samples (20 mL) were collected using venipuncture in plain serum tubes (Becton Dickinson vacutainer^®^ plus plastic serum tube, Franklin Lakes, NJ, USA). Blood samples were allowed to clot for 30–60 min at room temperature and centrifuged at 3000 rpm for 10 min at 4 °C. After centrifugation, sera were aliquoted into cryotubes for immediate storage at −80 °C.

### 4.3. RNA Isolation

Total RNA from 200 µL of each serum sample was extracted using the miRNeasy Serum/Plasma Kit (Qiagen, Venlo, The Netherlands). This was done according to the manufacturer’s recommendations, except for the following modifications: (A) A set of 3 proprietary spike-in controls (MiRXES, Singapore), representing high, medium, and low levels of RNA was added into the sample lysis buffer (QIAzol Lysis Reagent, Qiagen, Venlo, The Netherlands) prior to sample RNA isolation. The spike-in controls are 20-nucleotide RNAs with unique sequences (distinct from any of the 2588 annotated mature human miRNAs in miRBase version 21). These control RNAs are used to monitor RNA isolation efficiency and to normalize for technical variations during RNA isolation; (B) Bacteriophage MS2 RNA was added into sample lysis buffer (1 µg/mL ofQIAzol) to improve RNA isolation yield; (C) The samples were centrifuged at 18,000× *g* for 15 min at room temperature after mixing with chloroform; (D) RNA was eluted in 25 µL of RNase-free water.

### 4.4. RT-qPCR Detection of miRNA Expression

A tightly controlled RT-qPCR workflow was used to quantify the expression of miRNAs in each blood sample. Serum RNA was reverse-transcribed using miRNA-specific reverse transcription (RT) primers according to the manufacturer’s instructions (MiRXES) on a Veriti™ Thermal Cycler (Applied Biosystems, Waltham, MA, USA). Multiplexed RT reactions were performed using RT primers specific for each miRNA. For discovery, 324 RT primers were divided into 6 multiplex primer pools (50-plex to 60-plex per pool) to minimize non-specific crossovers and primer-primer interactions. For each RNA sample, we performed 6 multiplex RT reactions, each with 2 µL of isolated RNA. Synthetic templates for standard curves of each miRNA (6-log serial dilution of 107 to102 copies) and a non-template control (nuclease-free water spiked with MS2) were reverse-transcribed concurrently with the serum RNA samples.

We pre-amplified all cDNAs, including those from synthetic miRNA standards, using a 14-cycle PCR reaction with Augmentation Primer Pools (MiRXES) on the Veriti™ Thermal Cycler. Single qPCR was then performed on the amplified cDNA samples using a miRNA-specific qPCR assay and ID3EAL miRNA qPCR Master Mix according to the manufacturer’s instructions (MiRXES). The qPCR reactions were then performed with technical duplicates on the ViiA™ qPCR system (384-well configuration, Applied Biosystems).

Raw threshold cycle (Ct) values were calculated using the ViiA™ 7 RUO software with automatic baseline setting and a threshold of 0.5. RT-qPCR efficiency and potential cDNA amplification bias were assessed by analyzing the Ct values of the synthetic miRNA standards. The use of synthetic miRNA standard curves allowed us to absolutely quantify the copy numbers of miRNA expressed in each sample. Absolute expression of each miRNA (number of copies present) was calculated by interpolation of sample Ct values with synthetic miRNA standard curves after correcting for variations in RT-qPCR efficiency.

### 4.5. Biomarker Discovery

The global geometric mean normalization method was used to normalize the miRNA expression and identify miRNAs with statistically significant *p*-values and log2-transformed fold changes. The normalized miRNA expression values were used to compare the expression levels of individual miRNAs between malignant and benign breast lesions. Statistical significance of changes in miRNA expression was determined using the Student’s *t*-test. *p*-values were corrected for multiple hypothesis testing using the false discovery rate (FDR) adjustment [35]. We used FDR-adjusted *p*-value < 0.05 to identify miRNAs that were differentially expressed between malignant and benign lesions.

### 4.6. Biomarker Panel Building and Optimization

A two-fold cross-validation procedure, incorporating a feature selection algorithm and a logistic regression predictive model, was used to build and optimize miRNA biomarker panels in the Discovery Cohort. Samples were partitioned into equally-sized training and test sets for two-fold cross-validation. Prediction model performance was evaluated using the area under the curve (AUC) based on the receiver operating characteristics (ROC) curves. Two-hundred rounds of the two-fold cross-validation procedure were carried out for each biomarker panel comprising two to eight miRNAs. The sequential forward floating selection (SFFS) algorithm [36] was used to select miRNA biomarkers for inclusion in each biomarker panel. A logistic regression model was used to train predictive models for calculating a 6-miRNA biomarker score, which correlates with the probability of a patient being diagnosed with breast cancer given the expression levels of miRNAs included in the biomarker panel [37]. A higher biomarker score would mean a higher probability of the patient having breast cancer.

## 5. Conclusions

In conclusion, we have identified a circulating six-miRNA biomarker panel for the stratification of malignant and benign breast lesions in women with abnormal mammograms. Our findings highlight the potential use of circulating miRNAs for disease discrimination before histological diagnosis, which could be used in the future in conjunction with mammography. This warrants future studies with prospectively collected cohorts or blinded studies to evaluate the clinical utility of these circulating miRNAs for the detection of breast cancer.

## Figures and Tables

**Table 1:** Clinicopathological characteristics of patient cohort.

Characteristics	Discovery (*n* = 269)		Validation (*n* = 269)	
	Benign (*n* = 197)	Malignant (*n* = 72)	Benign (*n* = 196)	Malignant (*n* = 73)
Age (years):				
Mean	50.41	55.58	50.29	54.63
Median	50.00	55.00	50.00	56.00
Range	30–70	32–70	25–82	40–72
Race:				
Chinese	160	64	152	65
non-Chinese	37	8	44	8
Tumor stage:				
Stage 0	-	26	-	27
Stage 1	-	26	-	26
Stage 2	-	18	-	16
Stage 3	-	2	-	4
Tumor size:				
≤10 mm	-	20	-	20
11 to 20 mm	-	30	-	25
>20 mm	-	24	-	28
Unknown	-	3	-	2
Tumor grade:				
Grade 1	-	16	-	18
Grade 2	-	29	-	32
Grade 3	-	23	-	22
Unknown	-	4	-	1
Lymph node status:				
Positive	-	51	-	54
Negative	-	17	-	14
Unknown	-	4	-	5

**Table 2:** Optimal miRNA biomarker panel.

miRNA	Coefficient	*p*-Value	Log2(Fold Change)
hsa-miR-451a	1.84	0.0004	0.39
hsa-miR-195-5p	0.94	0.0001	0.41
hsa-miR-126-5p	0.45	0.01	0.17
hsa-miR-423-3p	0.13	0.40	−0.09
hsa-miR-192-5p	−0.49	0.57	−0.07
hsa-miR-17-5p	−2.36	0.10	0.10

**Table 3:** Performance of optimal 6-miRNA biomarker panel.

Performance Characteristic	High Specificity Biomarker Score Cut-Off	High Sensitivity Biomarker Score Cut-Off
Sensitivity	41.1% (35.2%–47.2%)	79.5% (74.0%–84.0%)
Specificity	89.8% (85.4%–93.1%)	62.2% (56.1%–68.0%)
PPV	60.0% (53.9%–65.9%)	43.9% (38.0%–50.1%)
NPV	80.4% (75.0%–84.9%)	89.1% (84.5%–92.5%)

## Data Availability

The data presented in this study are available on request from the corresponding author. The data are not publicly available due to privacy restrictions.
